# Activity of a Long-Acting Echinocandin (CD101) and Seven Comparator Antifungal Agents Tested against a Global Collection of Contemporary Invasive Fungal Isolates in the SENTRY 2014 Antifungal Surveillance Program

**DOI:** 10.1128/AAC.02045-16

**Published:** 2017-02-23

**Authors:** Michael A. Pfaller, Shawn A. Messer, Paul R. Rhomberg, Mariana Castanheira

**Affiliations:** a JMI Laboratories, North Liberty, Iowa, USA; b University of Iowa, Iowa City, Iowa, USA

**Keywords:** echinocandins, Candida, antifungal susceptibility

## Abstract

The activity of CD101 and comparator antifungal agents against 606 invasive fungal isolates collected worldwide during 2014 was evaluated using the Clinical and Laboratory Standards Institute (CLSI) method. All Candida albicans (*n* = 251), Candida tropicalis (*n* = 51), Candida krusei (*n* = 16), and Candida dubliniensis (*n* = 11) isolates were inhibited by ≤0.12 μg/ml of CD101 and were susceptible or showed wild-type susceptibility to the other echinocandins tested. Five C. glabrata isolates (*n* = 100) displayed CD101 MIC values of 1 to 4 μg/ml, had elevated MICs of caspofungin (2 to >8 μg/ml), anidulafungin (2 to 4 μg/ml), and micafungin (2 to 4 μg/ml), and carried mutations on *fks1* and *fks2*. Candida parapsilosis (*n* = 92) and Candida orthopsilosis (*n* = 10) displayed higher CD101 MIC values (ranges, 0.5 to 4 μg/ml and 0.12 to 2 μg/ml, respectively), and similar results were observed for the other echinocandins tested. Fluconazole resistance was noted among 11.0% of Candida glabrata isolates, 4.3% of C. parapsilosis isolates, and 2.0% of C. albicans and C. tropicalis isolates. The activity of CD101 against Aspergillus fumigatus (*n* = 56) was similar to that of micafungin and 2-fold greater than that of caspofungin but less than that of anidulafungin. These isolates had wild-type susceptibility to itraconazole, voriconazole, and posaconazole. The echinocandins had limited activity against Cryptococcus neoformans (*n* = 19). CD101 was as active as the other echinocandins against common fungal organisms recovered from patients with invasive fungal infections. The long half-life profile is very desirable for the prevention and treatment of serious fungal infections, especially in patients who can then be discharged from the hospital to complete antifungal therapy on an outpatient basis.

## INTRODUCTION

Despite the broad utilization of echinocandins to treat invasive candidiasis in critically ill hospitalized patients, clinical resistance to these agents remains uncommon, although both breakthrough infections and acquired resistance mutations have been noted in some species of Candida (1). Although the currently available echinocandins are highly efficacious and relatively safe to use in the treatment of invasive candidiasis and other invasive fungal infections (IFIs), they must be administered daily by intravenous infusion, potentially prolonging the hospitalization of patients undergoing therapy and often limiting their use to the inpatient setting (2). The availability of an echinocandin with activity that is comparable to the activities of echinocandins presently in use but with a pharmacokinetic profile that allows less frequent administration would alter the standard of care (i.e., echinocandin therapy) so that it could be more easily administered in both inpatient and outpatient settings (3).

CD101 (Cidara Therapeutics, Inc.) is a novel echinocandin antifungal agent that displays chemical stability in plasma, in aqueous solution, and at elevated temperatures and that also possesses a long-acting pharmacokinetic profile (4, 5). CD101 is being developed for once-weekly intravenous administration for the treatment of invasive candidiasis and candidemia (6). Less frequent dosing of this agent should facilitate shorter and more cost-effective hospital stays, improve compliance for outpatients, and provide more convenient outpatient prophylaxis or maintenance treatment regimens.

Surveillance of IFIs provides useful information concerning the patterns of etiologic agents and the emergence of resistance to both established and newly introduced antifungal agents (7, 8). The application of modern methods for species identification and characterization of antifungal resistance mechanisms provides a level of standardization and clarity that makes these observations useful in the ongoing fight against antifungal resistance (7, 9, 10).

Preliminary *in vitro* susceptibility data obtained using Clinical and Laboratory Standards Institute (CLSI) broth microdilution (BMD) methods for yeasts (11) and filamentous fungi (12) have demonstrated the excellent potency and spectrum of activity of CD101 against both Candida and Aspergillus spp. (3); however, determination of the activity of this agent in comparison with the activities of azoles and other echinocandins against an expanded array of Candida spp. and Aspergillus fumigatus is important in assessing the potential for the use of this novel echinocandin in the management of IFIs due to these opportunistic pathogens.

In the present study, we performed a broad assessment of the activity of CD101 in comparison with the activities of other echinocandins (anidulafungin, caspofungin, and micafungin), azoles (fluconazole, itraconazole, posaconazole, and voriconazole), and amphotericin B by testing a total of 606 isolates of Candida (531 isolates; 7 species), Cryptococcus neoformans var. grubii (19 isolates), and Aspergillus fumigatus sensu stricto (56 isolates) obtained during the 2014 SENTRY Antifungal Surveillance Program. All isolates were tested using CLSI BMD methods (11, 12).

## RESULTS AND DISCUSSION

The activity of CD101 and comparator agents against the CLSI-recommended quality control (QC) strains was tested at least 20 times during the surveillance program. QC results for the comparator agents were within established ranges, except for three values for micafungin that were within the expected range upon repeat testing. MIC values for CD101 were 0.03 to 0.06 μg/ml for Candida krusei ATCC 6258, 0.5 to 1.0 μg/ml for Candida parapsilosis ATCC 22019, and ≤0.008 μg/ml for both Aspergillus flavus ATCC 204304 and A. fumigatus MYA-3626.

Among the 606 fungal isolates tested, 531 (87.6%) were Candida spp., 19 (3.1%) were Cryptococcus neoformans var. grubii, and 56 (9.7%) were A. fumigatus sensu stricto (Table 1).

**TABLE 1:** Antifungal activity of CD101 and clinically available echinocandins against surveillance organisms/organism groups collected worldwide during 2014 and tested using the CLSI reference method

Organism (no. of isolates tested) and antifungal agent	No. (cumulative %) of isolates inhibited at MIC or MEC (μg/ml) of:												MIC or MEC (μg/ml)	
	≤0.008	0.015	0.03	0.06	0.12	0.25	0.5	1	2	4	8	>8	50%	90%
Candida albicans (251)														
CD101	17 (6.8)	107 (49.4)	91 (85.7)	28 (96.8)	8 (100.0)								0.03	0.06
Anidulafungin	69 (27.5)	101 (67.7)	54 (89.2)	22 (98.0)	5 (100.0)								0.015	0.06
Caspofungin	4 (1.6)	76 (31.9)	135 (85.7)	34 (99.2)	2 (100.0)								0.03	0.06
Micafungin	17 (6.8)	171 (74.9)	63 (100.0)										0.015	0.03
Candida glabrata (100)														
CD101		3 (3.0)	59 (62.0)	29 (91.0)	4 (95.0)	0 (95.0)	0 (95.0)	1 (96.0)	3 (99.0)	1 (100.0)			0.03	0.06
Anidulafungin	1 (1.0)	0 (1.0)	11 (12.0)	62 (74.0)	21 (95.0)	0 (95.0)	0 (95.0)	0 (95.0)	3 (98.0)	2 (100.0)			0.06	0.12
Caspofungin			38 (38.0)	51 (89.0)	6 (95.0)	0 (95.0)	0 (95.0)	0 (95.0)	2 (97.0)	0 (97.0)	3 (100.0)		0.06	0.12
Micafungin	1 (1.0)	73 (74.0)	21 (95.0)	0 (95.0)	0 (95.0)	0 (95.0)	0 (95.0)	0 (95.0)	3 (98.0)	2 (100.0)			0.015	0.03
Candida parapsilosis (92)														
CD101							22 (23.9)	35 (62.0)	34 (98.9)	1 (100.0)			1	2
Anidulafungin							4 (4.3)	28 (34.8)	44 (82.6)	16 (100.0)			2	4
Caspofungin						31 (33.7)	49 (87.0)	11 (98.9)	1 (100.0)				0.5	1
Micafungin							10 (10.9)	57 (72.8)	25 (100.0)				1	2
Candida tropicalis (51)														
CD101	2 (3.9)	27 (56.9)	16 (88.2)	6 (100.0)									0.015	0.06
Anidulafungin	5 (9.8)	27 (62.7)	18 (98.0)	1 (100.0)									0.015	0.03
Caspofungin		12 (23.5)	23 (68.6)	15 (98.0)	1 (100.0)								0.03	0.06
Micafungin	1 (2.0)	15 (31.4)	27 (84.3)	8 (100.0)									0.03	0.06
Candida krusei (16)														
CD101		3 (18.8)	11 (87.5)	2 (100.0)									0.03	0.06
Anidulafungin		1 (6.2)	6 (43.8)	9 (100.0)									0.06	0.06
Caspofungin				4 (25.0)	10 (87.5)	2 (100.0)							0.12	0.25
Micafungin				8 (50.0)	8 (100.0)								0.06	0.12
Candida dubliniensis (11)														
CD101			6 (54.5)	4 (90.9)	1 (100.0)								0.03	0.06
Anidulafungin		1 (9.1)	3 (36.4)	7 (100.0)									0.06	0.06
Caspofungin		1 (9.1)	5 (54.5)	5 (100.0)									0.03	0.06
Micafungin		3 (27.3)	8 (100.0)										0.03	0.03
Candida orthopsilosis (10)														
CD101					1 (10.0)	3 (40.0)	1 (50.0)	4 (90.0)	1 (100.0)				0.5	1
Anidulafungin						3 (30.0)	2 (50.0)	4 (90.0)	1 (100.0)				0.5	1
Caspofungin				1 (10.0)	3 (40.0)	3 (70.0)	3 (100.0)						0.25	0.5
Micafungin					2 (20.0)	2 (40.0)	3 (70.0)	3 (100.0)					0.5	1
Cryptococcus neoformans var. grubii (19)														
CD101											6 (31.6)	13 (100.0)	>8	>8
Anidulafungin												19 (100.0)	>8	>8
Caspofungin											4 (21.1)	15 (100.0)	>8	>8
Micafungin												19 (100.0)	>8	>8
Aspergillus fumigatus (56)														
CD101	23 (41.1)	29 (92.9)	4 (100.0)										0.015	0.015
Anidulafungin	30 (53.6)	21 (91.1)	5 (100.0)										≤0.008	0.015
Caspofungin		18 (32.1)	35 (94.6)	3 (100.0)									0.03	0.03
Micafungin	16 (28.6)	38 (96.4)	2 (100.0)										0.015	0.015

Table 1 summarizes the *in vitro* susceptibilities of the 606 tested isolates to CD101, anidulafungin, caspofungin, and micafungin. Although a recent report highlighted the lack of reproducibility of caspofungin MIC results when isolates were tested using CLSI reference broth microdilution methods (13), the results were included in this study. Caspofungin is an important in-class comparator for the echinocandins; furthermore, the results of the tests reported here were generated in a single laboratory, decreasing the issues with the reproducibility of the results. To reduce the impact of these results, we did not apply caspofungin interpretative criteria in Table 2.

**TABLE 2:** Activity of CD101 and comparator antifungal agents when tested using the CLSI method

Organism (no. of isolates tested) and antifungal agent	MIC or MEC_50_ (μg/ml)	MIC or MEC_90_ (μg/ml)	Range (μg/ml)	% isolates meeting the following CLSI criterion^*a*^:			% isolates meeting the following ECV^*b*^:	
				S	I/SDD	R	WT	NWT
C. albicans (251)								
CD101	0.03	0.06	≤0.008–0.12	—^*c*^	—	—	—	—
Anidulafungin	0.015	0.06	≤0.008–0.12	100.0	0.0	0.0	100.0	0.0
Caspofungin^*d*^	0.03	0.06	≤0.008–0.12	—	—	—	—	—
Micafungin	0.015	0.03	≤0.008–0.03	100.0	0.0	0.0	100.0	0.0
Fluconazole	≤0.12	0.25	≤0.12–>128	98.0	0.0	2.0	96.8	3.2
Posaconazole	0.03	0.06	≤0.008–>8	—	—	—	92.4	7.6
Voriconazole	≤0.008	0.015	≤0.008–>8	99.2	0.0	0.8	98.4	1.6
Amphotericin B	1	1	0.5–1	—	—	—	100.0	0.0
C. glabrata (100)								
CD101	0.03	0.06	0.015–4	—	—	—	—	—
Anidulafungin	0.06	0.12	≤0.008–4	95.0	0.0	5.0	95.0	5.0
Caspofungin^*d*^	0.06	0.12	0.03–>8	—	—	—	—	—
Micafungin	0.015	0.03	≤0.008–4	95.0	0.0	5.0	95.0	5.0
Fluconazole	8	64	0.25–128	—	89.0	11.0	89.0	11.0
Posaconazole	0.5	2	0.03–4	—	—	—	99.0	1.0
Voriconazole	0.12	1	0.015–4	—	—	—	88.0	11.0
Amphotericin B	1	1	0.5–2	—	—	—	100.0	0.0
C. parapsilosis (92)								
CD101	1	2	0.5–4	—	—	—	—	—
Anidulafungin	2	4	0.5–4	82.6	17.4	0.0	100.0	0.0
Caspofungin^*d*^	0.5	1	0.25–2	—	—	—	—	—
Micafungin	1	2	0.5–2	100.0	0.0	0.0	100.0	0.0
Fluconazole	1	2	0.25–128	95.7	0.0	4.3	95.7	4.3
Posaconazole	0.06	0.12	0.03–1	—	—		98.9	1.1
Voriconazole	0.015	0.03	≤0.008–2	95.7	1.1	3.3	95.7	4.3
Amphotericin B	1	1	0.5–1	—	—	—	100.0	0.0
C. tropicalis (51)								
CD101	0.015	0.06	≤0.008–0.06	—	—	—	—	—
Anidulafungin	0.015	0.03	≤0.008–0.06	100.0	0.0	0.0	100.0	0.0
Caspofungin^*d*^	0.03	0.06	0.015–0.12	—	—	—	—	—
Micafungin	0.03	0.06	≤0.008–0.06	100.0	0.0	0.0	100.0	0.0
Fluconazole	0.5	0.5	≤0.12–8	98.0	0.0	2.0	98.0	2.0
Posaconazole	0.06	0.12	0.015–0.25	—	—	—	98.0	2.0
Voriconazole	0.03	0.06	≤0.008–0.25	98.0	0.0	2.0	94.1	5.9
Amphotericin B	1	1	0.5–1	—	—	—	100.0	0.0
C. krusei (16)								
CD101	0.03	0.06	0.015–0.06					
Anidulafungin	0.06	0.06	0.015–0.06	100.0	0.0	0.0	100.0	0.0
Caspofungin^*d*^	0.12	0.25	0.06–0.25	—	—	—	—	—
Micafungin	0.06	0.12	0.06–0.12	100.0	0.0	0.0	100.0	0.0
Fluconazole	32	32	16–64	—	—	—	100.0	0.0
Posaconazole	0.25	0.5	0.03–0.5	—	—	—	100.0	0.0
Voriconazole	0.25	0.5	0.06–0.5	100.0	0.0	0.0	100.0	0.0
Amphotericin B	1	1	1–2	—	—	—	100.0	0.0
C. dubliniensis (11)								
CD101	0.03	0.06	0.03–0.12	—	—	—	—	—
Anidulafungin	0.06	0.06	0.015–0.06	—	—	—	100.0	0.0
Caspofungin^*d*^	0.03	0.06	0.015–0.06	—	—	—	—	—
Micafungin	0.03	0.03	0.015–0.03	—	—	—	100.0	0.0
Fluconazole	≤0.12	≤0.12	≤0.12–0.25	—	—	—	100.0	0.0
Posaconazole	0.03	0.06	0.03–0.06	—	—	—	100.0	0.0
Voriconazole	≤0.008	≤0.008	≤0.008	—	—	—	100.0	0.0
Amphotericin B	0.5	0.5	0.25–1	—	—	—	100.0	0.0
C. orthopsilosis (10)								
CD101	0.5	1	0.12–2	—	—	—	—	—
Anidulafungin	0.5	1	0.25–2	—	—	—	100.0	0.0
Caspofungin^*d*^	0.25	0.5	0.06–0.5	—	—	—	—	—
Micafungin	0.5	1	0.12–1	—	—	—	100.0	0.0
Fluconazole	1	2	0.5–32	—	—	—	90.0	10.0
Posaconazole	0.12	0.12	0.06–0.25	—	—	—	100.0	0.0
Voriconazole	0.03	0.06	0.015–0.5	—	—	—	90.0	10.0
Amphotericin B	0.5	1	0.5–1	—	—	—	100.0	0.0
C. neoformans var. grubii (19)								
CD101	>8	>8	8–>8	—	—	—	—	—
Anidulafungin	>8	>8	8–>8	—	—	—	—	—
Caspofungin^*d*^	>8	>8	8–>8	—	—	—	—	—
Micafungin	>8	>8	8–>8	—	—	—	—	—
Fluconazole	2	4	1–4	—	—	—	100.0	0.0
Posaconazole	0.12	0.25	0.03–0.25	—	—	—	100.0	0.0
Voriconazole	0.03	0.06	0.03–0.06	—	—	—	100.0	0.0
Amphotericin B	1	1	0.5–1	—	—	—	—	—
A. fumigatus (56)								
CD101	0.015	0.015	≤0.008–0.03	—	—	—	—	—
Anidulafungin	≤0.008	0.015	≤0.008–0.03	—	—	—	—	—
Caspofungin^*d*^	0.03	0.03	0.015–0.06	—	—	—	—	—
Micafungin	0.015	0.015	≤0.008–0.03	—	—	—	—	—
Itraconazole	0.5	1	0.25–1	—	—	—	100.0	0.0
Posaconazole	0.25	0.5	0.06–0.5	—	—	—	100.0	0.0
Voriconazole	0.25	0.5	0.12–1	—	—	—	100.0	0.0
Amphotericin B	2	2	0.5–2	—	—	—	100.0	0.0

a Clinical and Laboratory Standards Institute (CLSI) breakpoint criteria. S, susceptible; I/SDD, intermediate/susceptible dose dependent; R, resistant.

b WT, wild type; NWT, non-wild type.

c —, not available.

d Caspofungin interpretative criteria were omitted due to reproducibility issues when this compound was tested (13).

When examined by species, CD101 at ≤0.12 μg/ml inhibited 95% of Candida glabrata isolates and 100% of Candida albicans, Candida dubliniensis, Candida tropicalis, and C. krusei isolates. All isolates of C. parapsilosis and Candida orthopsilosis were inhibited by CD101 at ≤4 μg/ml, and all isolates of A. fumigatus were inhibited by ≤0.03 μg/ml (the minimum effective concentration [MEC] value). The activity of CD101 was comparable to that of the three echinocandin comparators against all species of Candida with the exception of C. krusei, where CD101 was 4-fold more active than caspofungin (MIC_50_ and MIC_90_, 0.03 and 0.06 μg/ml, respectively, for CD101 versus 0.12 and 0.25 μg/ml, respectively, for caspofungin). Whereas none of the echinocandins were active against C. neoformans var. grubii, all four agents were very active against the tested isolates of A. fumigatus (MEC for 90% of isolates tested [MEC_90_], 0.015 to 0.03 μg/ml).

When examined using either the clinical breakpoints or the epidemiological cutoff values (ECVs) established by CLSI for the echinocandins, azoles, and amphotericin B (14–17), the vast majority of isolates were susceptible and/or showed wild-type (WT) susceptibility to all of the tested agents (Table 2). Fluconazole resistance was noted among 11.0% of C. glabrata isolates, 4.3% of C. parapsilosis isolates, and 2.0% of C. albicans and C. tropicalis isolates. Given these results, it is reasonable to assume that the CD101 MIC distributions obtained for each species in this survey represent the WT susceptibility distributions for this new echinocandin when tested by the CLSI BMD method (Tables 1 and 2).

There were five isolates of C. glabrata for which the CD101 MICs were ≥1 μg/ml and the MICs of the other echinocandins were ≥2 μg/ml; one harbored a mutation on *fks1* hot spot region 1 (HS 1) encoding the S629P alteration, two carried alterations on *fks2* HS 1 (F659S and S663P), and two carried alterations on both *fks1* HS 1 (S629P) and *fks2* HS 1 (S663P) (data not shown).

### Summary and conclusions.

The data presented here expand upon our previous observations (3) and demonstrate that the activity of CD101 is comparable to that of anidulafungin, caspofungin, and micafungin against a predominantly WT collection of Candida spp. and A. fumigatus isolates. Although only five isolates (all C. glabrata) were found to have non-WT (NWT) susceptibility to the echinocandins and harbored mutations in *fks1* and *fks2*, the results were consistent with our previous observations indicating that *fks* mutant strains of Candida exhibit elevated MIC values for CD101 as well as for the established echinocandins. On the basis of these data, it appears that MICs of ≤0.12 μg/ml for C. albicans, C. glabrata, C. dubliniensis, C. tropicalis, and C. krusei, MICs of ≤4.0 μg/ml for C. parapsilosis and C. orthopsilosis, and an MEC of ≤0.03 μg/ml for A. fumigatus define the upper limit of the WT MIC distributions for CD101 and the common species of Candida and Aspergillus. Further evaluation against less common species of yeasts and molds and expanded clinical development of this long-acting echinocandin are warranted.

## MATERIALS AND METHODS

### Organisms.

A total of 606 nonduplicate fungal isolates prospectively collected during 2014 from 38 medical centers located in North America (161 isolates; 10 sites), Europe (294 isolates; 17 sites), the Asia-Pacific region (82 isolates; 6 sites), and Latin America (69 isolates; 5 sites) were evaluated. The isolates selected were from the following sources: bloodstream (379 strains), normally sterile body fluids, tissues, and abscesses (22 strains), respiratory tract specimens (96 strains), and other or nonspecified body sites (109 strains). The yeast isolates were subcultured and screened using CHROMagar Candida (Becton Dickinson, Sparks, MS) to ensure purity and to differentiate Candida albicans from Candida dubliniensis, Candida tropicalis, and C. krusei. Isolates suspected to be either C. albicans or C. dubliniensis (green colonies on CHROMagar) were incubated at 45°C. All other yeast and all mold isolates were submitted to matrix-assisted laser desorption ionization–time of flight mass spectrometry (MALDI-TOF MS) using a MALDI Biotyper system according to the manufacturer's instructions (Bruker Daltonics, Billerica, MA). Three isolates that were not identified by either phenotypic or proteomic methods were identified using sequencing-based methods as previously described (8, 10, 18).

### Antifungal susceptibility testing.

All isolates were tested by BMD according to CLSI methods outlined in documents M27-A3, M38-A2, and M27-S4 (11, 12, 14). The systemically active antifungal agents tested were CD101, anidulafungin, caspofungin, micafungin, fluconazole, itraconazole, posaconazole, voriconazole, and amphotericin B. The ranges of the antifungal agent concentrations tested were 0.008 to 16 μg/ml for the echinocandins, amphotericin B, itraconazole, and voriconazole and 0.12 to 128 μg/ml for fluconazole. MIC results were determined visually after 24 h (Candida spp.), 48 h (A. fumigatus), or 72 h (C. neoformans) of incubation at 35°C and were considered to be the lowest concentration of drug that resulted in ≥50% inhibition of growth relative to that of the growth control (azoles and echinocandins versus yeasts) or complete (100%) inhibition (itraconazole, posaconazole, and voriconazole versus A. fumigatus and amphotericin B versus both yeasts and molds). The echinocandin minimum effective concentration (MEC) values for A. fumigatus were determined as described in CLSI document M38-A2 (12). CLSI clinical breakpoints for echinocandins, fluconazole, and voriconazole were used for the five most common species of Candida (C. albicans, C. glabrata, C. parapsilosis, C. tropicalis, and C. krusei). Epidemiological cutoff values (ECV) were applied when available for the other species tested (14, 18).

### QC.

Quality control (QC) was ensured by testing the following strains recommended by CLSI: C. krusei ATCC 6258, C. parapsilosis ATCC 22019, A. flavus ATCC 204304, and A. fumigatus MYA-3626.

### Screening for 1,3-β-d-glucan synthase mutations.

All isolates of Candida spp. that were either resistant or that had non-wild-type (NWT) susceptibility (MIC > ECV) to one or more of the echinocandins were characterized for the presence or absence of a mutation in the hot spot (HS) regions of *fks1* and *fks2* (C. glabrata only) as described previously (19).
